# A porous metal-organic framework with ultrahigh acetylene uptake capacity under ambient conditions

**DOI:** 10.1038/ncomms8575

**Published:** 2015-06-30

**Authors:** Jiandong Pang, Feilong Jiang, Mingyan Wu, Caiping Liu, Kongzhao Su, Weigang Lu, Daqiang Yuan, Maochun Hong

**Affiliations:** 1 State Key Laboratory of Structure Chemistry, Fujian Institute of Research on the Structure of Matter, Chinese Academy of Sciences, Fuzhou, Fujian 350002, China; 2 University of Chinese Academy of Sciences, Beijing 100049, China; 3 Department of Chemistry, Texas A&M University, College Station, Texas 77843, USA

## Abstract

Acetylene, an important petrochemical raw material, is very difficult to store safely under compression because of its highly explosive nature. Here we present a porous metal-organic framework named **FJI-H8**, with both suitable pore space and rich open metal sites, for efficient storage of acetylene under ambient conditions. Compared with existing reports, **FJI-H8** shows a record-high gravimetric acetylene uptake of 224 cm^3^ (STP) g^−1^ and the second-highest volumetric uptake of 196 cm^3^ (STP) cm^−3^ at 295 K and 1 atm. Increasing the storage temperature to 308 K has only a small effect on its acetylene storage capacity (∼200 cm^3^ (STP) g^−1^). Furthermore, **FJI-H8** exhibits an excellent repeatability with only 3.8% loss of its acetylene storage capacity after five cycles of adsorption–desorption tests. Grand canonical Monte Carlo simulation reveals that not only open metal sites but also the suitable pore space and geometry play key roles in its remarkable acetylene uptake.

## Abstract (web summary)

The efficient storage of high-energy gaseous fuels is problematic due to the risk of explosion. Here, the authors design and employ a metal-organic framework composed of Cu(II) and an octacarboxylate ligand, which is able to store acetylene in high density but at atmospheric pressure.

## Graphical abstract

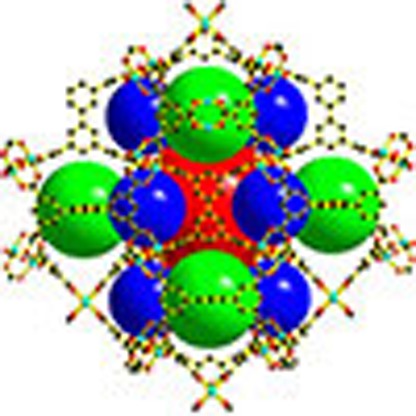

Acetylene is a very important chemical feedstock for modern industry^1^,^2^. Many widely used polymer products such as polyurethane and polyester plastics are synthesized from acetylene. However, the safe storage and transportation of acetylene still remain challenging because of its explosiveness when compressed under pressures over 2 atm at room temperature^3^,^4^. Therefore, acetylene gas extensively used in industry so far has to be stored in special cylinders filled with acetone and porous materials suffering from lower acetylene purity and higher storage cost. Fortunately, the emergence of porous metal-organic frameworks (MOFs) brings promising solutions to the above problem due to their excellent performance for storage and separation of gases such as H_2_, O_2_, CH_4_ and CO_2_ (refs 5, 6, 7, 8, 9, 10, 11, 12, 13, 14, 15, 16, 17). MOFs have been recently studied for acetylene storage application^18^,^19^,^20^,^21^,^22^,^23^,^24^,^25^,^26^,^27^. For example, at 273 K ZJU-5 shows a high acetylene uptake of 290 cm^3^ (STP) g^−1^. However, the uptake drastically decreases to 193 cm^3^ (STP) g^−1^ when the temperature rises to 295 K^18^. To effectively improve acetylene storage capacity at room temperature, Chen *et al.* explored a series of microporous MOFs with different structures and porosities and concluded that open Cu(II) sites and suitable pore space in MOFs played crucial roles for acetylene storage^18^,^19^,^20^,^21^,^22^. In addition, the dendritic multi-carboxylate ligands with *m*-benzenedicarboxylate moieties tend to form various polyhedral nanocages along with rich open Cu(II) sites, which has been demonstrated as an efficient approach to improve the gas uptakes^26^,^27^,^28^,^29^,^30^,^31^,^32^,^33^.

Considering the previous studies, we designed and synthesized a new robust multi-carboxylate ligand 3,3′,5,5′-tetra(3,5-dicarboxyphenyl)-4,4′-dimethoxy-biphenyl (H_8_tddb, Supplementary Fig. 1). Reaction of H_8_tddb and Cu(NO_3_)_2_ under solvothermal conditions resulted in a porous MOF ([Cu_4_(tddb)·(H_2_O)_4_]_*n*_·(solvent)_*x*_, abbreviated as **FJI-H8**) with both suitable pore space and open metal sites. At 295 K, **FJI-H8** exhibits a record-high acetylene uptake of 224 cm^3^ (STP) g^−1^, greatly exceeding the previous record of 201 cm^3^ (STP) g^−1^ held by HKUST-1 (ref. 19). Increasing the storage temperature to 308 K has only small effect on its acetylene storage capacity (∼200 cm^3^ (STP) g^−1^). Furthermore, the acetylene adsorption amount of **FJI-H8** at 295 K has no obvious loss after five cycles of adsorption–desorption test.

## Results

### Structure of FJI-H8

**Figure 1:**
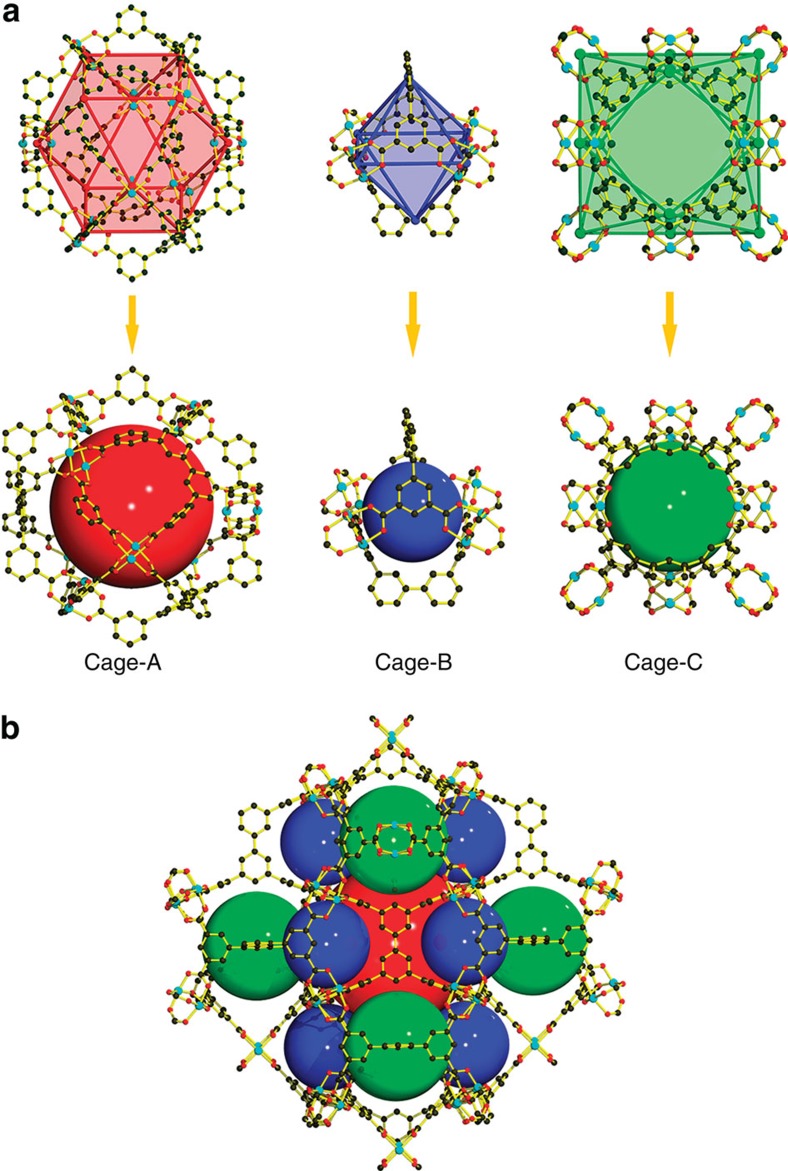
Structural representations of FJI-H8 from X-ray diffraction data. (**a**) Three types of polyhedral nanocages observed in **FJI-H8**, that is, one regular cuboctahedral cage (Cage-A), one distorted octahedral cage (Cage-B) and one distorted cuboctahedral cage (Cage-C). (**b**) Combination of the three types of polyhedral nanocages. (The hydrogen atoms and hydroxymethyl groups are omitted for clarity.)

Single-crystal X-ray diffraction experiments revealed that **FJI-H8** crystallized in the tetragonal space group *P*4_2_/*nnm* (Supplementary Data 1). In the asymmetric unit, there are one-quarter of organic ligand and two kinds of Cu(II) ions both with the occupancies of 50%. One metal site is located on a mirror plane, while the other resides on a twofold axis. Further, the dinuclear core is centred about an inversion site. All the eight carboxyl groups are deprotonated in the organic ligand. Two inner phenyl rings of tddb are coplanar, while the four outer isophthalate groups are almost perpendicular to the diphenyl with a dihedral angle of 78.85° (Supplementary Fig. 2). Each tddb ligand coordinates to eight dicopper(II) paddlewheel secondary building units (SBUs) and each Cu_2_ SBU links to four tddb ligands. As anticipated, there are three types of polyhedral nanocages in **FJI-H8** (Supplementary Fig. 3), that is, one regular cuboctahedron (Cage-A), one distorted octahedron (Cage-B) and one distorted cuboctahedron (Cage-C) (Fig. 1a). Cage-A is constructed by eight Cu_2_ SBUs and four tddb ligands. The centres of the eight paddlewheels together with the centroids of the four tddb ligands are considered as the 12 four-connected vertices of the cage. Therefore, the pore diameter is around 15 Å, which is estimated through the separations of two opposite vertexes. In addition, eight open Cu(II) sites point towards the centre of the cage and can interact directly with gas molecules residing inside, which can improve the gas adsorption ability^34^,^35^,^36^,^37^,^38^. Cage-B consists of four Cu_2_ SBUs and two halves of tddb ligands, with a dimension of ∼8 Å. For Cage-C, its 12 vertices consist of the centroids of eight Cu_2_ SBUs and four halves of tddb ligands, respectively. In addition, the dimension of this cage is estimated to be around 12 Å. On the whole, Cage-A is linked by six Cage-C through six rhombic faces and eight Cage-B through eight triangular faces (Supplementary Fig. 4). Similarly, Cage-C is linked by six Cage-A through six rhombic faces and eight Cage-B by sharing eight *m*-benzenedicarboxylate moieties (Supplementary Fig. 5). However, Cage-B is linked by four Cage-A through four triangular faces and four Cage-C by sharing four *m*-benzenedicarboxylate moieties (Supplementary Fig. 6). It should be noted that there are two kinds of Cu_2_ SBUs in **FJI-H8** due to the fact that the distance between the paddlewheels and the centroids of the ligands are slightly different (8.96 and 9.45 Å, respectively). For the sake of clarity, if we simplify two types of Cu_2_ SBUs as two kinds of four-connected nodes and the tddb ligands as eight-connected nodes, **FJI-H8** adopts the rare (4,4,8)-c URJ network with the topological point symbol of 4^14^.6^12^.8^2^ (Supplementary Fig. 7)^39^.

### Porosity and N_2_ adsorption of FJI-H8

**Figure 2:**
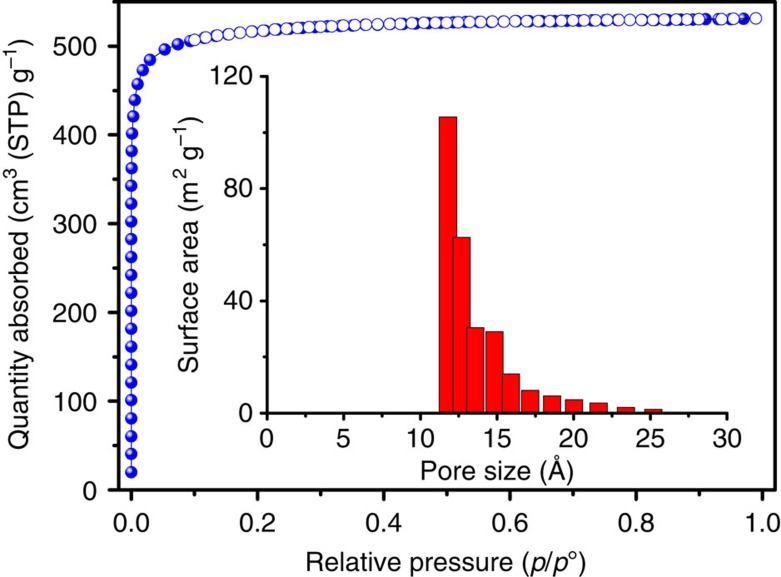
N_2_ sorption data for FJI-H8. N_2_ sorption isotherm at 77 K (filled symbols: adsorption; open symbols: desorption); inset: pore size distribution analysed by NLDFT method.

The solvent accessible volume in fully evacuated **FJI-H8** is 62.4% calculated by PLATON with a probe of 1.8 Å (ref. 40). To check the permanent porosity of **FJI-H8**, nitrogen adsorption isotherm was measured at 77 K and 1 atm. As demonstrated by powder X-ray diffraction (PXRD, Supplementary Fig. 8), the activated sample retained the crystallinity after being evacuated for 10 h under 80 ^o^C. The N_2_ sorption of **FJI-H8** exhibits a typical reversible type I isotherm with a saturated adsorption amount of 531 cm^3^ (STP) g^−1^, indicating the microporous nature of **FJI-H8**. The Brunauer–Emmett–Teller apparent surface area calculated from the N_2_ adsorption data is 2025±15 m^2^ g^−1^ and is well consistent with theoretical one (1907, m^2^ g^−1^) calculated by Poreblazer^41^, which demonstrates that the sample is fully activated. Accordingly, the total pore volume is 0.82 cm^3^ g^−1^. From the N_2_ adsorption data, analysis by the non-local density functional theory (NLDFT) model confirms a narrow distribution of micropores around 12 Å (Fig. 2).

### Acetylene adsorption property of FJI-H8

**Figure 3:**
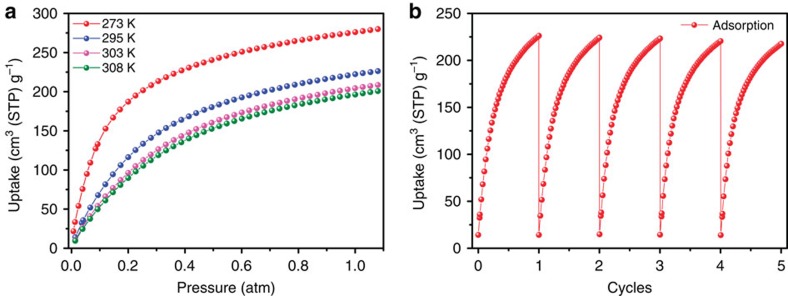
C_2_H_2_ adsorption properties. (**a**) C_2_H_2_ adsorption isotherms of **FJI-H8** at 273, 295, 303 and 308 K. (**b**) Cycles of C_2_H_2_ adsorption for **FJI-H8** at 295 K.

Considering the open metal sites and moderate pores in **FJI-H8**, its low-pressure acetylene uptake was measured under 1 atm. As expected, the C_2_H_2_ adsorption amount for **FJI-H8** reaches up to 277 cm^3^ (STP) g^−1^ at 273 K and 1 atm, slightly less than the record of 290 cm^3^ (STP) g^−1^ (ref. 18). In practice, C_2_H_2_ gas is stored at ambient temperatures. Therefore, the C_2_H_2_ adsorption experiment at room temperature (295 K) was carried out. Exhilaratingly, **FJI-H8** exhibits an adsorption amount of 224 cm^3^ (STP) g^−1^ for acetylene at 295 K and 1 atm, which is greatly higher than those of the two famous MOFs, known as HKUST-1 (201 cm^3^ (STP) g^−1^) and CoMOF-74 (197 cm^3^ (STP) g^−1^) (Table 1). Surprisingly, **FJI-H8** exhibits an acetylene uptake of 206 cm^3^ (STP) g^−1^ at 303 K and 1 atm, which prompts us to investigate its acetylene uptake at an even higher temperature. The most commendable aspect is that the acetylene adsorption amount of **FJI-H8** still reached 200 cm^3^ (STP) g^−1^ even when the temperature increased to 308 K (Fig. 3a). This value is comparable to that of HKUST-1 at 295 K. In other words, the acetylene uptake capacity of **FJI-H8** decreases by a rate of 2.2 cm^3^ g^−1^ K^−1^ with the experimental temperature increasing from 273 to 308 K, which is almost only half to that of ZJU-5 (3.9 cm^3^ g^−1^ K^−1^) from 273 K to 298 K (Supplementary Table 1). Therefore, **FJI-H8** is more suitable for practical applications over a wide temperature range around room temperature. In consideration of its practical application, we also tested the repeatability of **FJI-H8** for acetylene storage. About 100 mg of desolvated sample was loaded onto an ASAP2020-M analyser and five cycles of acetylene adsorption at 295 K were recorded without the reactivation process between each cycle. For **FJI-H8**, there is only a 3.8% loss in absorbed quantity of C_2_H_2_ after five cycles, which indicates that **FJI-H8** is promising in refillable acetylene storage (Fig. 3b)^42^.

The isosteric heat of acetylene adsorption for **FJI-H8** is calculated to be 32.0 kJ mol^−1^ based on the C_2_H_2_ adsorption isotherms at 273, 295, 303 and 308 K (Supplementary Fig. 9). This value is larger than that of HKUST-1 (30.4 kJ mol^−1^). As has been reported previously, the high density of open metal sites within MOF materials may play crucial roles in the high acetylene storage capacities and high adsorption enthalpies. Supposing each open metal site binds one acetylene molecule, as established by Chen *et al.*, the acetylene uptake by open Cu(II) sites in HKUST-1 accounts for c.a. 60% of the total acetylene uptake at room temperature. For CoMOF-74, almost 80% of the total acetylene uptake is contributed by open Co(II) sites assuming each Co(II) site binds one acetylene molecule. Moreover, for MgMOF-74, if all the open Mg(II) sites can be fully loaded with one acetylene molecule per one open Mg(II) site, the theoretical acetylene uptake by open metal sites is even larger than the experimentally measured value at room temperature. As listed in Table 1, for most MOF materials, acetylene uptakes by open metal sites account for almost half of the acetylene uptake or even higher. However, in **FJI-H8**, the open Cu(II) site density is 3.59 mmol g^−1^, which is lower than those for the reported MOFs with high acetylene uptake capacities. On the whole, the open Cu(II) sites can only contribute 87 cm^3^ of the total 224 cm^3^ for the acetylene storage capacity at 295 K and 1 atm. Thus, the remaining acetylene storage capacity should come from the suitable pore space in **FJI-H8**. It should be noted that the acetylene uptake by the pore space accounts for >60% of the whole acetylene uptake in **FJI-H8**, which is rarely seen in other reported MOFs. More surprisingly, the whole acetylene uptake decreases to 200 cm^3^ (STP) g^−1^ with a slight loss of 24 cm^3^ (STP) g^−1^, when the temperature rises from 295 to 308 K. The above result indicates relatively strong interactions between acetylene molecules and pore space in **FJI-H8**.

In general, there are two kinds of representations to measure the gas adsorption properties of adsorbent materials, that is, gravimetric capacity in the unit of cm^3^ (STP) g^−1^ and volumetric capacity in the unit of cm^3^ (STP) cm^−3^. The relationship between them is *V*_volumetric_=*V*_gravimetric_ × *ρ*. If we take the solvent-free crystal density into consideration, **FJI-H8** shows an acetylene uptake of 196 cm^3^ (STP) cm^−3^ at 295 K and 1 atm, which is only inferior to the uptake of 230 cm^3^ (STP) cm^−3^ for CoMOF-74 under the same conditions^20^. On the basis of the volumetric acetylene uptake, **FJI-H8** shows a safe acetylene storage density of 0.23 g cm^−3^ in bulk material at 295 K and 1 atm, which is equivalent to the value of an imaginary state of acetylene under 21.3 MPa at room temperature and is ∼100 times of the compression limit for the safe storage of acetylene (0.2 MPa) at room temperature^3^,^43^,^44^. This value is also at the highest level for the reported MOFs, and is only slightly lower than that of CoMOF-74 (0.27 g cm^−3^), which is consistent with the volumetric analysis (Table 2).

Similarly, supposing each open metal site binds one acetylene molecule, the acetylene uptake by open Cu(II) sites in HKUST-1 accounts for c.a. 54% of the volumetric acetylene uptake at room temperature. For CoMOF-74, 73% of the volumetric acetylene uptake is contributed by open Co(II) sites if each Co(II) site binds one acetylene molecule. Much like the gravimetric capacity, for the volumetric acetylene uptake of MgMOF-74 if all the open Mg(II) sites can be fully loaded with one acetylene molecule per each open Mg(II) site, the theoretical acetylene uptake by open metal sites is even larger than the experimental one at room temperature. As listed in Table 2 for most MOF materials, the volumetric acetylene uptake capacities contributed by open metal sites account for almost half of the acetylene uptakes or even higher. However, in **FJI-H8**, the open Cu(II) sites can only contribute 70 cm^3^ out of the total 196 cm^3^ volumetric acetylene storage capacity at 295 K and 1 atm. Comparatively, the volumetric acetylene uptake by the suitable pore space in **FJI-H8** contributed to 64% of the whole amount, which is consistent with the gravimetric analysis aforementioned.

### The GCMC simulation

**Figure 4:**
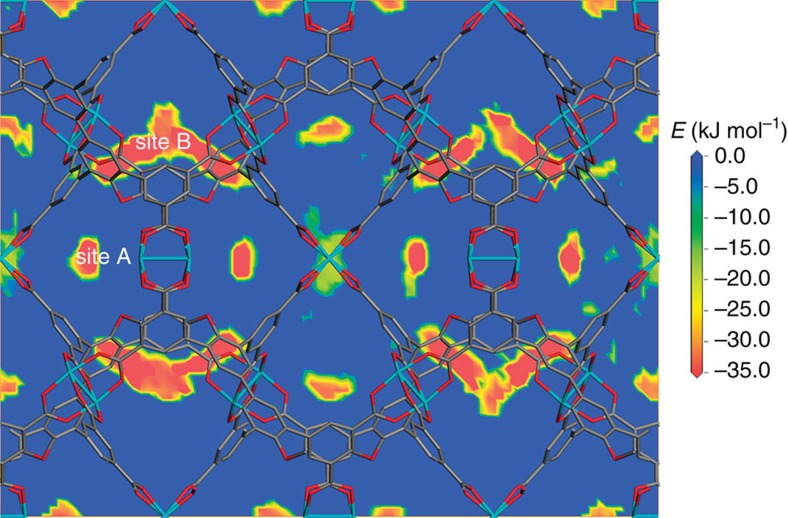
Slice through the calculated potential field for acetylene in FJI-H8. The slice is viewed along the crystallographic *a* axis. The framework is displayed in capped sticks plot, and the hydrogen atoms are omitted for clarity.

Theoretically, the confirmation of acetylene adsorption sites within the MOF skeletons is very important for us to design new MOFs-based gas storage materials. The most intuitive method to get the confirmation of adsorbed acetylene molecules is single-crystal diffractions or the poly-crystal power diffractions^45^,^46^. However, the above methods usually require high quality crystalline samples with high stability, which is not always available. On the other hand, theoretical simulation is a powerful tool that can give us a lot of useful information^19^,^47^,^48^. To understand the acetylene-framework interactions, the acetylene adsorption property of **FJI-H8** was studied by grand canonical Monte Carlo (GCMC) simulations^45^,^46^,^47^,^48^. The calculated C_2_H_2_ adsorption isotherm is shown in Supplementary Fig. 10. As expected, the agreement between simulation and experiment of acetylene adsorption is almost perfect at pressures below 0.3 atm, whereas the uptake at higher pressures is slightly overestimated with a deviation of 7%. Furthermore, the calculated adsorption heat of 28.7 kJ mol^−1^ is also comparable to the experimental one (Supplementary Fig. 11). Slice through the calculated potential field for acetylene is displayed in Fig. 4. Two preferential adsorption regions are showed in the field. As anticipated, the highest potential values are located around the unsaturated metal centres. Surprisingly, a strong increase of the interaction potential is visible in the small Cage-B, which is surrounded by eight benzene rings. However, it is noteworthy that the adsorption behaviour in Cage-B should be attributed to the interactions between the adsorbed acetylene molecules and the surrounding benzene rings rather than the four misaligned open copper sites. The adsorption in the octahedral pore is observed at the entrance windows to this pore, which is similar to HKUST-1 (refs 19, 47). Therefore, the ultrahigh acetylene uptake of **FJI-H8** can be attributed to the suitable pore space together with open metal sites.

## Discussion

To our knowledge, the open metal sites within MOFs materials usually play an important role in the high gas storage capacities due to the strong interactions between acetylene molecules and the metal sites. Supposing each open metal site binds one acetylene molecule, for most previously reported MOF materials the acetylene uptake by open metal sites theoretically accounts for over 60% of the whole acetylene uptake at room temperature or even higher. However, in **FJI-H8**, the open Cu(II) site density is low and the acetylene uptake by the pore space accounts for >60% of the whole amount, which is rarely seen in other reported MOFs. As is to be expected, the acetylene uptake by the pore space accounts for >60% of the whole amount even when the crystal density is taken into consideration (Table 2). Ideally, the perfect adsorbent materials should have both high gravimetric uptake and high volumetric capacity. In practice, very few adsorbents including MOFs can meet this requirement. For example, CoMOF-74 shows the highest volumetric acetylene uptake of 230 cm^3^ (STP) cm^−3^ at 295 K and 1 atm; however, the gravimetric acetylene uptake of CoMOF-74 is only 197 cm^3^ (STP) g^−1^ (ref. 20). Similarly, although the gravimetric acetylene capacity of HKUST-1 reaches up to 201 cm^3^ (STP) g^−1^ at 295 K and 1 atm, it shows a volumetric acetylene uptake of 177 cm^3^ (STP) cm^−3^ (ref. 19). In our case, compared with the reported results measured at 295 K and 1 atm, **FJI-H8** exhibits the highest acetylene uptake of 224 cm^3^ (STP) g^−1^ in gravimetric capacity, and also shows a high value of 196 cm^3^ (STP) cm^−3^ in terms of volumetric capacity, which is only lower than that of CoMOF-74, due to its lower crystal density. More importantly, high acetylene uptakes around room temperature, low decrease rate of acetylene uptakes in the temperature range from 273 to 308 K and excellent repeatability make **FJI-H8** a suitable candidate for practical applications. Although MOF-based acetylene storage has not exhibited economical advantage to acetone-based one at this stage, the purity of acetylene stored in **FJI-H8** is higher than that in acetone. Furthermore, since acetone is an explosive pollutant, the storage of acetylene by recyclable **FJI-H8** is safer and cleaner. More importantly, the cost of MOF-based acetylene storage may decrease markedly if large-scale application is implemented. Therefore, the exploration of MOFs for safe and pure acetylene storage is significant not only for theoretical studies but also for practical applications.

In conclusion, **FJI-H8** is a promising candidate for acetylene storage at around room temperature. The high acetylene uptake of **FJI-H8** shows that not only open metal sites but also suitable pore space plays key roles in MOF-based acetylene storage. Our results shed light on the rational design and synthesis of new MOFs materials for acetylene storage based on the two factors above.

## Methods

### Materials and equipment

All reagents and solvents used in synthetic studies were commercially available and used as supplied without further purification. The ligand H_8_tddb (H_8_tddb=3,3′,5,5′-tetra(3,5-dicarboxyphenyl)-4,4′-dimethoxy-biphenyl) was synthesized through the routine in the Supplementary Fig. 1.

Elemental analyses for C, H and N were carried out on a German Elementary Vario EL III instrument. ^1^H NMR spectra were obtained on a Burker AVANCE 400 (400 MHz) for spectrometer. Mass-accurate match spectra were obtained using a DECAX-30000 LCQ Deca XP mass spectrometer with electro-spray ionization (ESI). The PXRD patterns were collected by a Rigaku Mini 600 X-ray diffractometer using Cu Kα radiation (*λ*=1.54 Å). Simulations of the PXRD spectrum are carried out by the single-crystal data and diffraction-crystal module of the Mercury program available free of charge via internet at http://www.ccdc.cam.ac.uk/products/mercury/.

*Synthesis of 3,3′,5,5′-tetrabromo-4,4′-biphenol (1)*. Bromine (13.8 ml, 268.6 mmol) was rapidly added to a solution of 4,4′-biphenol (10 g, 54 mol) in methanol (400 ml). After 1 h of stirring, the resulting precipitate was filtered and washed sequentially with aqueous solutions of NaHCO_3_, Na_2_SO_3_ and water. The resulting white powder was dissolved in acetone and dried over anhydrous Na_2_SO_4_. Pure compound **1** was obtained by recrystallization in acetone (14.6 g, 54%). ^1^H NMR (400 MHz, CDCl_3_) *δ* 5.93 (s, 2H), 7.60 (s, 4H) p.p.m.

*Synthesis of 3,3′,5,5′-tetrabromo-4,4′-dimethoxy-1,1′-biphenyl (2)*. Compound **1** (4.0 g, 8 mmol), iodomethane (6.8 g, 48 mmol) and K_2_CO_3_ (3.3 g, 24 mmol) were dissolved into acetonitrile (100 ml). The reaction mixture was heated at reflux for 18 h and then cooled to room temperature. Acetonitrile was removed using rotary evaporator and the resulting mixture was poured into water and extracted with dichloromethane (3 × 100 ml). The combined organic layers were dried over anhydrous MgSO_4_, and then the solvent was removed again using rotary evaporator. After purification by column chromatography on silica gel using hexane as eluent and evaporation of the fraction containing the product, compound **2** was obtained as a white powder. (2.88 g, 68%). ^1^H NMR (400 MHz, CDCl_3_) *δ* 3.94 (s, 6H), 7.65 (s, 4H) p.p.m.

*Synthesis of 3,3′,5,5′-tetra(diethyl-3,5-dicarboxyphenyl)-4,4′-dimethoxy-biphenyl (3)*. Compound **2** (1.06 g, 2 mmol), diethyl 5-(4,4,5,5-tetramethyl-1,3,2-dioxaborolan-2-yl)-1,3-benzenedicarboxylate (4.18 g, 12 mmol), Cs_2_CO_3_ (11.8 g, 36 mmol) and tetrakis(triphenylphosphine)palladium (0.092 g, 0.08 mmol) were added to a 500-ml schlenk flask charged with stir bar. The flask was pumped under vacuum and refilled with N_2_ for three times, and then 350 ml degassed 1,4-dioxane was transferred to the system and the reaction mixture was heated to 85 ^o^C for 72 h under N_2_ atmosphere. After the reaction mixture was cooled to room temperature, the organic solvent was removed using rotary evaporator, and the resulting mixture was poured into water and extracted with dichloromethane (3 × 100 ml). The combined organic layers were dried over anhydrous MgSO_4_, and then the solvent was removed again using rotary evaporator. After purification by column chromatography on silica gel using ethyl acetate/hexane (1:5 v/v) as eluent and evaporation of the fraction containing the product, compound **3** was obtained as a pale yellow solid. (1.60 g, 73%). ^1^H NMR (400 MHz, CDCl_3_): *δ* 1.44 (t, 24H), 3.21 (s, 6H), 4.45 (q, 16H), 7.66 (s, 4H), 8.52 (s, 8H), 8.73 (s, 4H) p.p.m.

*Synthesis of 3,3′,5,5′-tetra(3,5-dicarboxyphenyl)-4,4′-dimethoxy-biphenyl (H_8_tddb).* Compound **3** (2.19 g, 4 mmol) was dissolved in 20 ml of THF, to which 20 ml of 10 M NaOH aqueous solution was added. The mixture was stirred under reflux for 10 h, and then the organic solvent was removed using rotary evaporator. The aqueous phase was acidified to pH 2 using 6 M HCl aqueous solution. The resulting precipitate was collected via filtration, washed with water (200 ml) and dried under vacuum to afford **H**_**8**_**tddb**. (1.58 g, 91%). ^1^H NMR (400 MHz, DMSO-*d*_6_) *δ*, 3.10 (s, 6H), 7.97 (s, 4H), 8.43 (s, 8H), 8.51 (s, 4H), 13.40 (s, 8H) p.p.m. ^13^C NMR (100 MHz, DMSO-*d*_6_) *δ*, 167.0, 154.5, 139.0, 136.4, 134.5, 134.4, 132.0, 130.1, 129.4, 61.1 p.p.m. ESI-MS (ESI^−^ mode): calculated for C_46_H_30_O_18_: 869.1. Found: 869.0.

*Synthesis of FJI-H8*. Cu(NO_3_)_2_·2H_2_O (30 mg) and H_8_tddb (10 mg) were dissolved in 1.5 ml of *N*,*N*-diethylformamide and 0.45 ml of water in a 25 ml pyrex vial, to which 25 μl of HCl were added. The mixture was heated in 85 °C oven for 12 h to yield 6 mg of blue–green crystals (yield: 46% based on H_8_tddb). The crystals obtained were filtered and washed with *N*,*N*-diethylformamide. Elemental analyses calculated (%) for C_46_H_42_O_26_Cu_4_. (After activation and absorbed small amount of water, the crystal has a formula of Cu_4_(H_2_O)_4_(tddb)·6H_2_O): C 42.63, H 3.26; found: C 45.82, H 3.65.)

### X-ray data collection and structure determination of FJI-H8

A blue–green block crystal of **FJI-H8** was taken directly from the mother liquor, transferred to oil and mounted into a loop. The crystal was kept at 100.0(1) K during data collection on a SuperNova diffractometer equipped with Cu-Kα radiation (*λ*=1.5418 Å) using a *ω* scan mode. The crystal structure was solved by direct method and refined by full-matrix least squares on *F*^2^ using SHELXTL package^49^. All non-hydrogen atoms were refined with anisotropic displacement parameters. The hydrogen atoms on the aromatic rings were located at geometrically calculated positions and refined by riding. However, the hydrogen atoms for the coordinated molecules cannot be found from the residual electron density peaks and the attempt of theoretical addition was not done. Therefore, the number of reported hydrogen atoms is more than the calculated one. The free solvent molecules are highly disordered in **FJI-H8**, and attempts to locate and refine the solvent peaks were unsuccessful. The diffused electron densities resulting from these solvent molecules were removed using the SQUEEZE routine of PLATON^40^; structures were then refined again using the data generated. Crystal data are summarized in Supplementary Table 2.

### Low-pressure gas sorption measurements

Low-pressure (<1 bar) adsorption measurements were performed using a Micromeritics ASAP 2020-M surface area and pore size analyser. The fresh crystalline sample of **FJI-H8** was degassed under dynamic vacuum at 80 ^o^C for 10 h after solvent exchange with methanol and then dichloromethane for 3 days each. A colour change from blue–green to deep purple–blue was observed during the activation process, which is attributed to the remove of terminal coordinated water of dicopper(II) paddlewheel SBUs, thus indicating the generation of open metal sites in the framework. Low-pressure N_2_ adsorption isotherms were measured at 77 K in a liquid nitrogen bath. The specific surface areas were determined using the Brunauer–Emmett–Teller model from the N_2_ sorption data. Low-pressure acetylene adsorption isotherms were measured at 273, 295, 303 and 308 K. The isosteric heat of adsorption was calculated through the Clausius–Clapeyron equation using the four sets of acetylene adsorption data collected.

### Computational methods

To characterize the adsorption sites of acetylene molecules in **FJI-H8**, GCMC simulations were carried out using Sorption Module in Materials Studio^50^. The **FJI-H8** framework was fixed at the crystallographic data based on the single-crystal X-ray diffraction. Four unit cells of **FJI-H8** (2 × 2 × 1) were used to construct the simulation box of the GCMC run. Then, the structural parameters of simulation box are *a*=*b*=35.8514 Å and *c*=28.0627 Å, as well as *α*=*β*=*γ*=90.

*DFT calculations*. Density functional theory (DFT) calculations were performed to derive the charges to be used in the GCMC simulations to estimate the adsorption isotherms of acetylene in **FJI-H8**. The atomic coordinates were taken from the experimental crystallographic data. The cluster included building units (for example, metal ion nodes and the organic linker) representative of the unit cells. Details of structure and atom types of the **FJI-H8** clusters are shown in Supplementary Fig. 12. The DFT calculations were performed with the Gaussian 09 (ref. 51) software at the B3LYP/6-31G* level of theory. Partial atomic charges were extracted using the ChelpG method^52^ by fitting them to reproduce the electrostatic potential generated by the DFT calculations. The charge of Cu was slightly adjusted to result in a neutral framework. Resulting partial charges for **FJI-H8** are given in Supplementary Table 3.

*Acetylene model*. The model of acetylene molecule was taken from the literature^47^. In this model, the acetylene molecule is a rigid structure where the C–C and C–H bond lengths are fixed at 1.2111 and 1.0712 Å, respectively (Supplementary Fig. 13). To account for the electrostatic interaction, point charges of 0.2780 *e* were assigned to H and C atoms of acetylene molecule, which were derived from DFT calculations using the ESP methods. Supplementary Table 4 shows bond lengths, ESP charge *q* and quadrupole moment *θ* for acetylene. To represent van der Waals interactions, the acetylene molecule was treated as a two-site model, in which H atoms in acetylene molecule were represented as non-interacting atoms and the Lennard–Jones (LJ) positions located on the carbon atoms. The LJ parameters for *sp*-hybridized carbon was taken from the central CH groups of 2-butene in the literature^53^.

*Force-field parameters*. LJ parameters for **FJI-H8** atoms were taken from the Universal force field^54^. LJ parameters for acetylene and LJ parameters representing the interaction of the acetylene molecules with the copper centres of **FJI-H8** were taken from the literature^47^. Supplementary Table 5 shows the LJ parameters for all atom types found in **FJI-H8** and acetylene.

*GCMC simulation*. These simulations were performed with the Sorption module of Materials Studio50. All GCMC simulations included a 4,000,000-cycle equilibration period followed by a 4,000,000-cycle production run. Atoms in **FJI-H8** were held fixed at their crystallographic positions. The van der Waals interactions were represented using a LJ potential, applying Lorentz–Berthelot mixing rules to calculate interactions between different atom types. An LJ cutoff distance of 12.5 Å was used for the simulations. The Ewald sum technique was used to compute the electrostatic interactions. Four unit cells of **FJI-H8** were used for the simulations. Acetylene isotherms were simulated at 295 K up to 1.0 bar. GCMC simulations reported the absolute adsorption data, which were then used to compute the excess adsorption data for comparison with experimental data using the relation


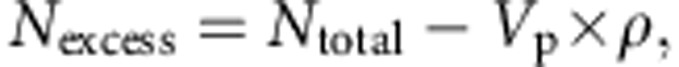


where *ρ* is the bulk density of acetylene at simulation conditions. The density needed was calculated using the Peng–Robinson equation of state. *V*_p_ is the pore volume calculated by PLATON^40^.

## Additional information

**Accession codes**: The X-ray crystallographic coordinates for structure reported in this Article have been deposited at the Cambridge Crystallographic Data Centre (CCDC), under deposition number CCDC 1048132. These data can be obtained free of charge from The Cambridge Crystallographic Data Centre via www.ccdc.cam.ac.uk/data_request/cif.

**How to cite this article:** Pang, J. *et al.* A porous metal-organic framework with ultrahigh acetylene uptake capacity under ambient conditions. *Nat. Commun.* 6:7575 doi: 10.1038/ncomms8575 (2015).

## Supplementary Material

Supplementary Figures and Supplementary Tables Supplementary Figures 1-13 and Supplementary Tables 1-5

Supplementary Data 1 Crystal structure data

## Figures and Tables

**Table 1:** Contributions of open metal sites (OMSs) and pore space in acetylene uptakes for selected MOFs at room temperature and 1 atm for gravimetric capacity in the unit of cm^3^ (STP) g^−1^ *.

**Material**	**OMS density (mmol g**^−**1**^)	**C**_**2**_**H**_**2**_ **uptake (cm**^**3**^ **(STP) g**^−**1**^)		
		**By OMS**	**By pore space**	**Sum**
**FJI-H8**	3.59	87	137	224
**FJI-H8**†	3.59	87	113	200
HKUST-1 (ref. 19)	4.96	120	81	201
CoMOF-74 (ref. 20)	6.41	155	42	197
ZJU-5 (ref. 18)	3.87	95	98	193
MgMOF-74 (ref. 20)	8.24	199‡	—	184
NOTT-101 (ref. 55)	3.44	84	100	184
ZJU-7 (ref. 56)	3.46	85	95	180
Cu-TDPAT (ref. 26)	3.74	91	87	178
PCN-16 (ref. 55)	4.19	102	74	176

^*^The OMS density of MOFs was calculated based on the crystal information files.

^†^Data for **FJI-H8** at 308 K.

^‡^The value of C_2_H_2_ uptake by OMS is larger than the sum value maybe because of the interaction between open Mg(II) sites and acetylene molecules are so weak that open Mg(II) sites cannot be fully loaded.

**Table 2:** Contributions of open metal sites (OMSs) and pore space in acetylene uptakes for selected MOFs at room temperature and 1 atm for volumetric capacity in the unit of cm^3^ (STP) cm^−3^ *.

**Material**	**Framework Density (g cm**^−**3**^)†	**OMS density (mmol cm**^−**3**^)	**C**_**2**_**H**_**2**_ **uptake (cm**^**3**^ **(STP) cm**^−**3**^)			**Density**‡ **(g cm**^−**3**^)	**P**§ **(MPa)**
			**By OMS**	**By pore space**	**Sum**		
**FJI-H8**	0.873	3.13	70	126	196	0.23	21.3
**FJI-H8**||	0.873	3.13	70	105	175	0.20	19.9
HKUST-1 (ref. 19)	0.879	4.36	97	80	177	0.21	19.3
CoMOF-74 (ref. 20)	1.169	7.49	168	62	230	0.27	25.1
ZJU-5 (ref. 18)	0.598	2.31	52	63	115	0.13	12.5
MgMOF-74 (ref. 20)	0.909	7.49	168¶	—	167	0.19	18.2
NOTT-101 (ref. 55)	0.684	2.35	53	73	126	0.15	13.9
ZJU-7 (ref. 56)	0.750	2.60	58	77	135	0.16	14.7
Cu-TDPAT (ref. 26)	0.783	2.93	66	73	139	0.16	15.1
PCN-16 (ref. 55)	0.723	3.03	68	59	127	0.15	13.8

^*^The OMS density of MOFs was calculated based on the crystal information files.

^†^The framework density was calculated from single-crystal X-ray data.

^‡^Density of adsorbed C_2_H_2_ in bulk material.

^§^Pressure of C_2_H_2_ at 295 K corresponding to the calculated density of adsorbed C_2_H_2_ in bulk material.

^||^Data for **FJI-H8** at 308 K.

^¶^The value of C_2_H_2_ uptake by OMS is larger than the sum value maybe because of the interaction between open Mg(II) sites and acetylene molecules are so weak that open Mg(II) sites cannot be fully loaded.
