# Supplementary material for: A porous metal-organic framework with ultrahigh acetylene uptake capacity under ambient conditions
Source: Nat Commun. 2015 Jun 30;6:7575. doi: 10.1038/ncomms8575 (PMC4491824; doi:10.1038/ncomms8575)
Supplement: Supplementary Figures and Supplementary Tables — Supplementary Figures 1-13 and Supplementary Tables 1-5 [file ncomms8575-s1.pdf]

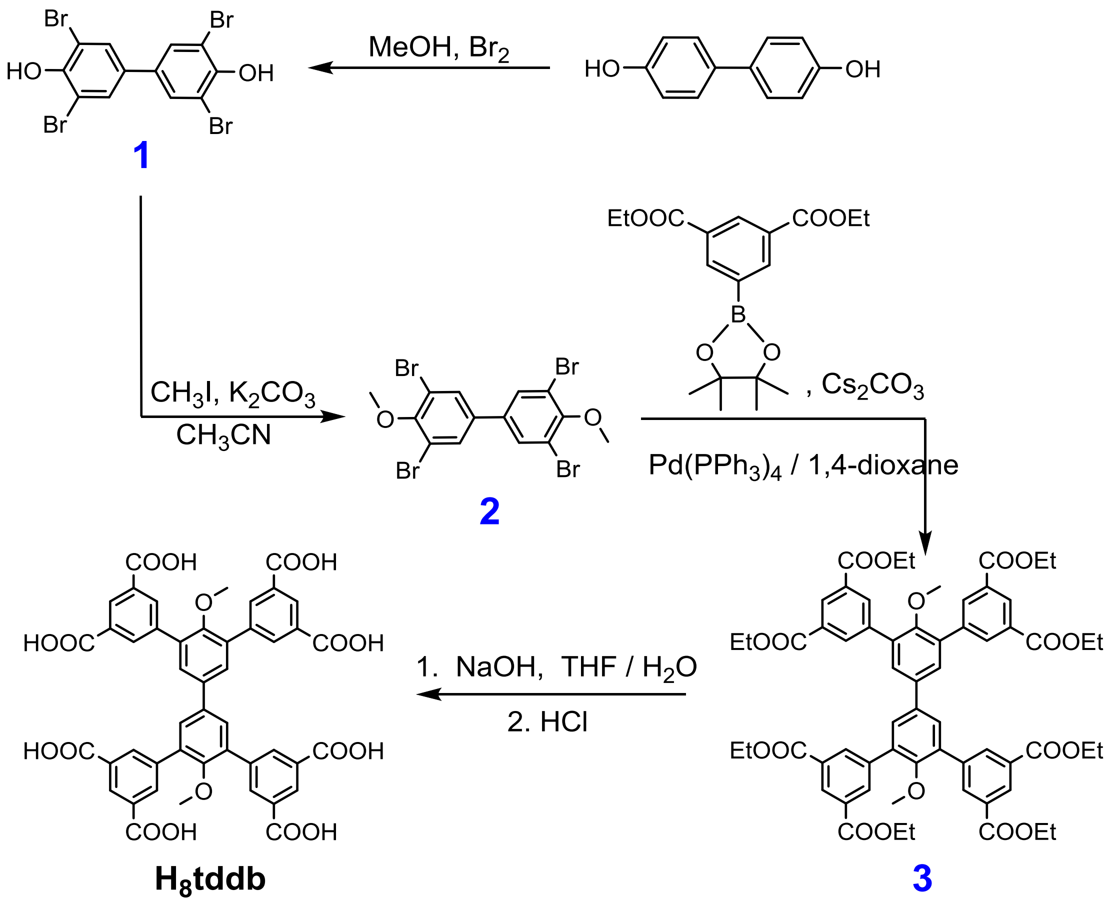

Supplementary Figure 1 | Synthesis scheme of the ligand H$_8$tddb

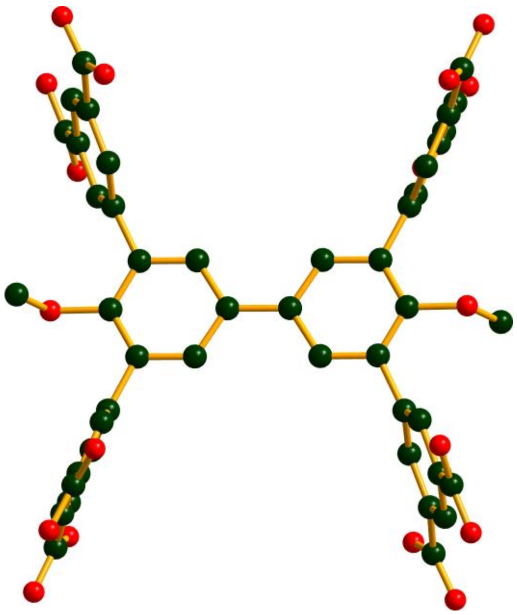

Supplementary Figure 2 | The steric configuration of the ligand tddb^{8-} in **FJI-H8** (The hydrogen atoms are omitted for clarity).

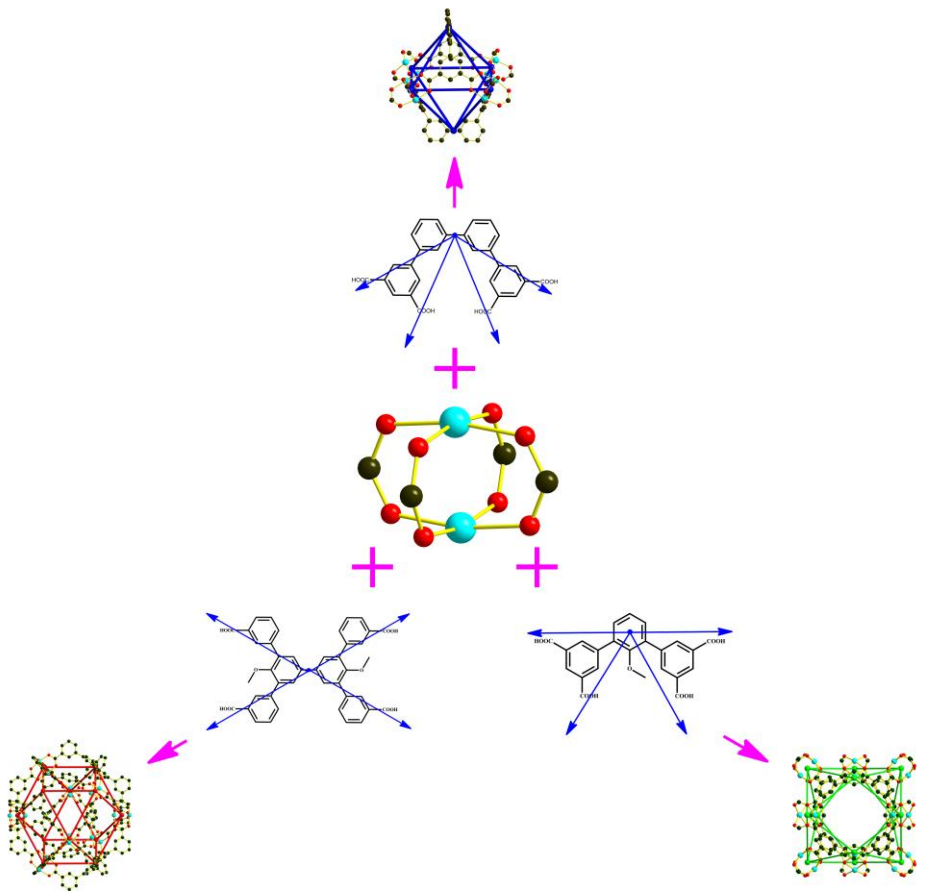

Supplementary Figure 3 | Different simplified 4-connected nodes for three types of nanocages in **FJI-H8** (The hydrogen atoms and hydroxymethyl groups are omitted for clarity).

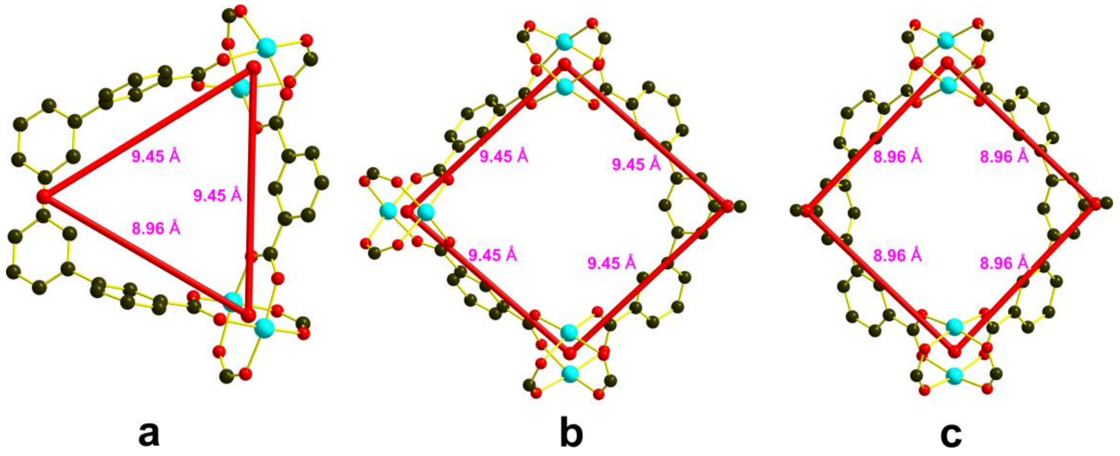

Supplementary Figure 4 | Three kind of faces observed in Cage-A. **(a)**: The triangular face; **(b)** and **(c)**: two kind of rhombic faces (The hydrogen atoms and hydroxymethyl groups are omitted for clarity).

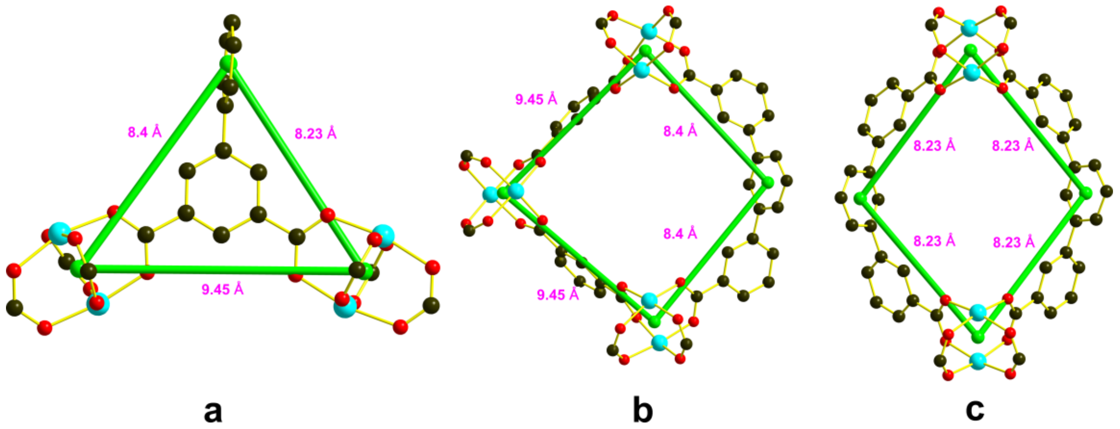

Supplementary Figure 5 | Three kind of faces observed in Cage-C. **(a)**: The triangular face; **(b)** and **(c)**: two kind of rhombic faces (the hydrogen atoms and hydroxymethyl groups are omitted for clarity).

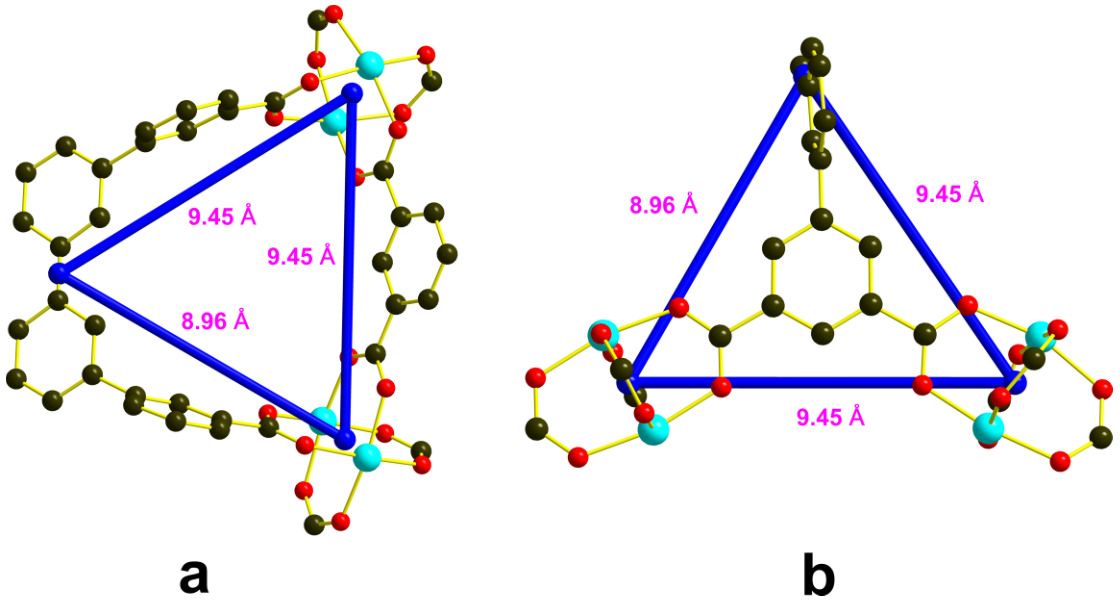

Supplementary Figure 6 | Two kind of triangular faces observed in Cage-B (the hydrogen atoms hydroxymethyl groups are omitted for clarity).

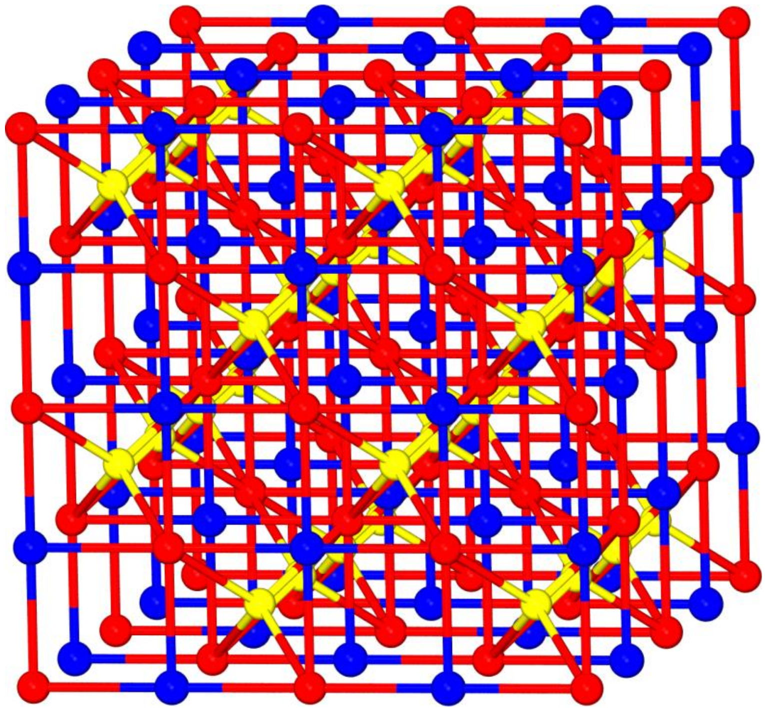

Supplementary Figure 7 | Topology of **FJI-H8** simplified the Cu_2 SBUs as 4-connected nodes and the tddb ligands as 8-connected nodes.

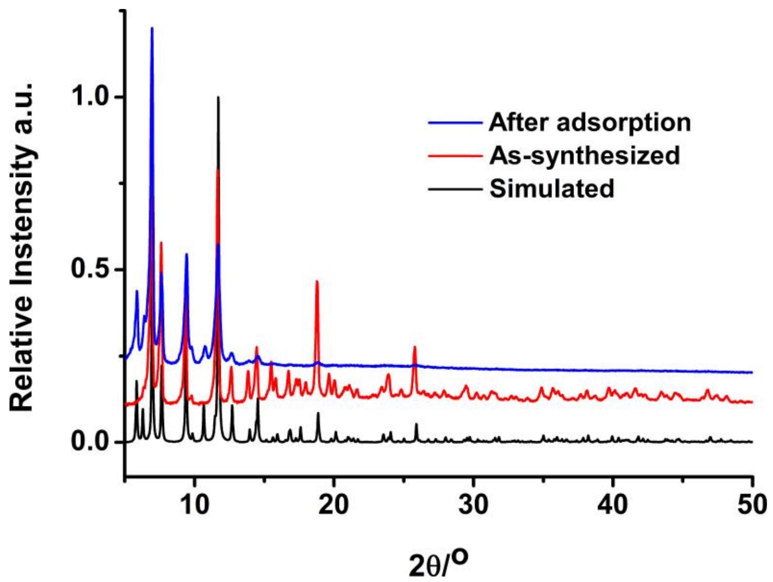

Supplementary Figure 8 | Powder X-ray diffraction (PXRD) patterns of **FJI-H8**: pattern simulated from single-crystal structure in black, experimental pattern for the as-synthesized sample in red and that for the sample after adsorption in blue.

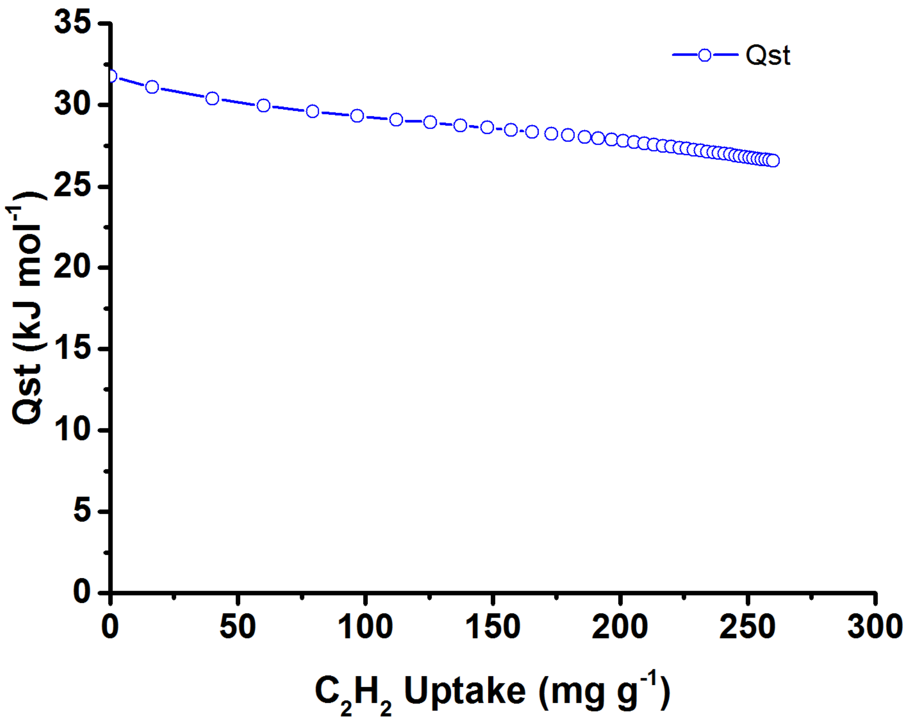

Supplementary Figure 9 | Isosteric heat of C_2H_2 adsorption at low coverage for **FJI-H8**.

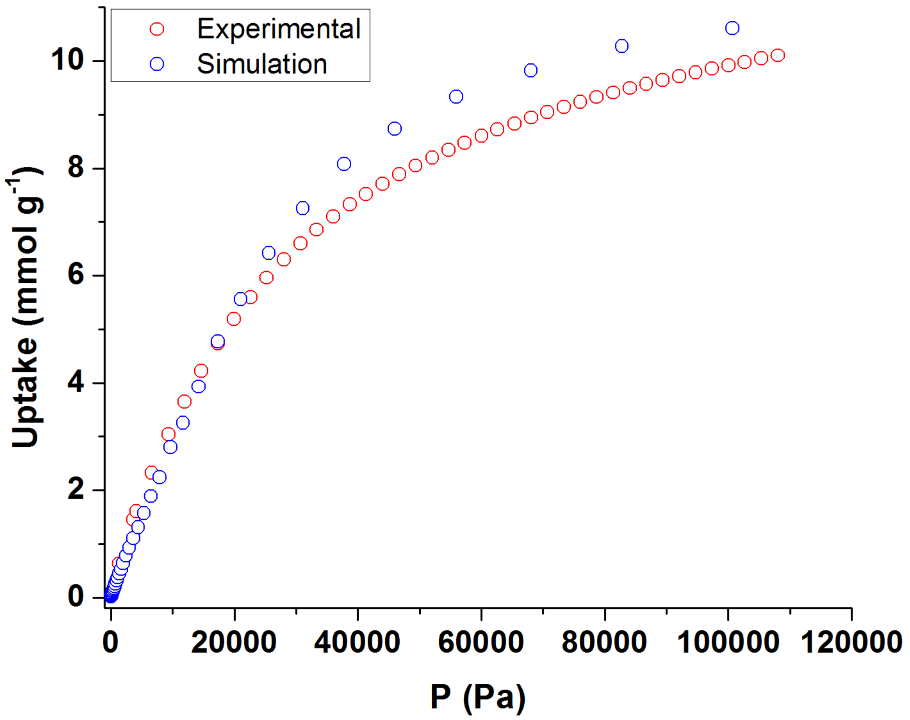

Supplementary Figure 10 | The simulated adsorption isotherm and the experimental one for C_2H_2 of **FJI-H8** at 295 K.

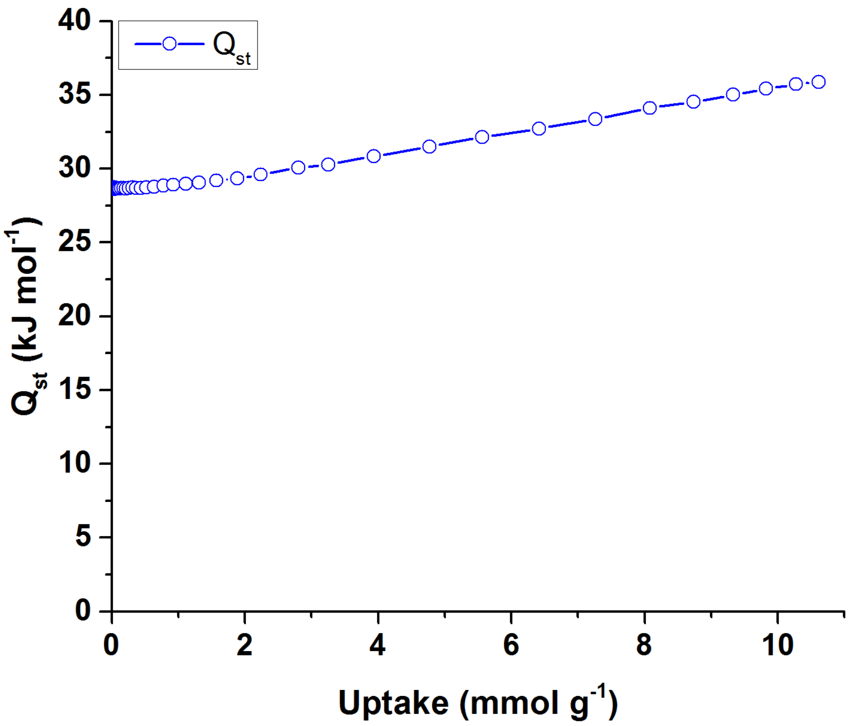

Supplementary Figure 11 | The simulated adsorption enthalpy for C_2H_2 of **FJI-H8**.

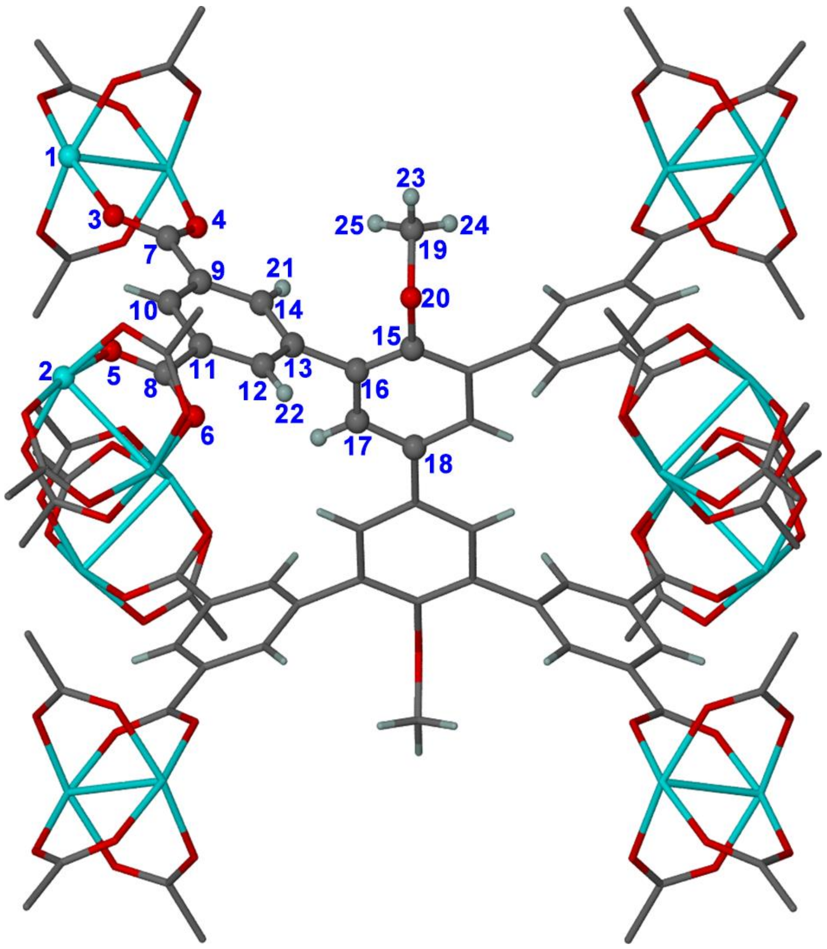

Supplementary Figure 12 | Cluster used for deriving partial charges on atoms in **FJI-H8**.

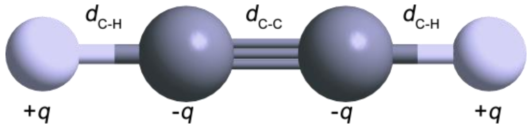

Supplementary Figure 13 | Acetylene molecule including designators of bond lengths and partial charges.

Supplementary Table 1 | The decrease rate of acetylene uptake amounts with the increase of the experimental temperatures for selected MOFs materials.

Material	Temperature Range (K)	Decrease Rate (cm^3 g^{-1} K^{-1})
**FJI-H8**	273 – 295	2.4
**FJI-H8**	295 – 308	1.8
**FJI-H8**	273 – 308	2.2
**HKUST-1**	273 – 295	2.2
**CoMOF-74**	273 – 295	1.3
**ZJU-5**	273 – 298	3.9
**MgMOF-74**	273 – 295	1.1
**NOTT-101**	273 – 296	3.6
**ZJU-7**	273 – 298	2.6
**Cu-TDPAT**	273 – 298	2.8
**PCN-16**	273 – 298	3.5

Supplementary Table 2. Crystal data and structure refinement for **FJI-H8**

Identification code	FJI-H8
Empirical formula	$C_{184}H_{120}Cu_{16}O_{88}$
Formula weight	4755.60
Moiety formula	$[Cu_4(tddb)•(H_2O)_4]_n•(solvent)_x$
Temperature (K)	100.0(1)
Wavelength (Å)	1.54184
Crystal system	tetragonal
Space group	$P4_2/nnm$
a (Å)	17.9257(3)
c (Å)	28.0627(7)
Volume (Å^3)	9017.4(4)
Z	16
Density (calculated) (g cm^{-3})	0.876
Absorption coefficient (mm^{-1})	1.452
F(000)	2392
Crystal size (mm^3)	0.1×0.05×0.05
θ range for data collection (º)	3.814 to 67.927
Limiting indices	-20<= *h* <= 21, -12 <= *k* <= 21, -34<= *l* <= 34
Reflections collected / unique	18677 / 4709 [*R*(int) = 0.0387]
Completeness	98.5 %
Absorption correction	multi-scan
Data/restraints/params	18677/0/174
Refinement method	Full-matrix least squares on F^2
Goodness of fit on F^2	1.002
Final *R* indices [I>2σ(I)]	*R*1 = 0.0488, *wR*2 = 0.1206
R indices (all data)	*R*1 = 0.0648, *wR*2 = 0.1255

Supplementary Table 3 | ESP charges calculated for FJI-H8.

**Atom No**	1	2	3	4	5
**Charge (e)**	1.007 (Cu)	1.038 (Cu)	-0.631 (O)	-0.631 (O)	-0.633 (O)
**Atom No**	6	7	8	9	10
**Charge (e)**	-0.650 (O)	0.759 (C)	0.766 (C)	-0.040 (C)	-0.128 (C)
**Atom No**	11	12	13	14	15
**Charge (e)**	-0.064 (C)	-0.195 (C)	0.257 (C)	-0.224 (C)	0.301 (C)
**Atom No**	16	17	18	19	20
**Charge (e)**	-0.068 (C)	-0.240 (C)	0.089 (C)	0.182 (C)	-0.149 (O)
**Atom No**	21	22	23	24	25
**Charge (e)**	0.154 (H)	0.151 (H)	0.010 (H)	0.018 (H)	0.018 (H)

Supplementary Table 4 | Bond lengths, ESP charge q, and quadrupole moment θ for acetylene

d_{C-C} (Å)	d_{C-H} (Å)	q (e)	θ (e Å^2)
1.2111	1.0712	0.2780	1.359

Supplementary Table 5 | Lennard-Jones parameters representing framework atoms and acetylene.

	H	C	O	Cu	C in C_2H_2	Cu-C(C_2H_2)
R_0 (Å)	2.8860	3.8510	3.5000	3.4950	4.2654	2.7664
D_0 (kJ mol^{-1})	0.1841	0.4393	0.2510	0.0209	0.4812	4.6831
